# Notes and News

**Published:** 1906-03-31

**Authors:** 


### Notes and News.

The Viscount Portman has accepted the office of President of the Samaritan Free Hospital in succession to the late Lord Leigh.

Worcester Infirmary benefits to the extent of £2,000 under the will of the late Mrs. Mary Day, of Lulsley, and Brompton Hospital for Consumptives to the extent of £500.

At the weekly meeting of the managers of the Edinburgh Royal Infirmary it was stated that Consols to the value of £5,265 had been transferred to the name of the Royal Infirmary from the estate of the late Dr. W. W. Allan.

It is stated that the Government propose, at the instance of Mr. Herbert Gladstone, Home Secretary, to appoint a Committee to inquire into and report upon the whole question of vivisection. The Anti-Vivisection Society anticipate that one of its members will be asked to join the Committee.

A welcome attempt is being made to establish closer relations between the Universities of Paris and London. With this end in view it is intended to establish a system of lectures at the University of London by Parisian professors, and lectures in the Paris University by University of London lecturers.

The famous amateur cricketer, Mr. V. E. Walker, who was also a millionaire, left a number of generous bequests to charities, including £2,000 each to the London Hospital and the Cancer Hospital, £1,000 each to the Middlesex Hospital, the City of London Hospital for Diseases of the Chest, the Royal Hospital for Incurables, the Poplar Hospital for Accidents, the Earlswood Idiot Asylum, the Brompton Hospital for Consumption and Diseases of the Chest, and the Victoria Hospital for Sick Children.

The Joint Committee for the removal of King's College Hospital to South London have received a cheque for £200, being the third instalment of the grant of £1,000 from the Clothworkers' Company.

At a meeting held last week at Leathersellers' Hall, London, in connection with the Women's Total Abstinence Union, Miss A. W. Richardson, who presided, said that drinking amongst women in this country was becoming the main social problem of the day. The Hon. Mrs. Eliot Yorke also spoke, and mentioned as an interesting fact that drunkenness was practically unknown amongst the women of Germany.

A girl of fifteen died at Plymouth last week in consequence of a misadventure at a science lecture at which a demonstration was given of a method of testing the alkalinity of a caustic soda solution. The unfortunate pupil, in pursuing the process, drew some of the solution into her mouth and swallowed it. An antidote was at once administered, but her mouth and throat were badly burnt, and though an operation was performed, she died from the effects.

In the annual report just issued by the Committee of Management of the British Ophthalmic Hospital at Jerusalem it is stated that there was a sudden and very large increase in the attendances of out-patients, especially during the ophthalmic season from July to December, chiefly from townspeople, and especially the Jews. It is hoped that a meeting of subscribers may be held during the present season, in order to call public attention to the necessity of further financial support for the institution which is doing such useful work among the Jews and natives of Syria.

At a meeting held on Wednesday, last week, at 76 Wimpole Street, W., to discuss the establishment of a new hospital for gentlewomen, Sir Wroth Lethbridge, who presided, said that the objects of the proposed institution, which would be in the nature of a surgical hostel, would be to provide a home where gentlewomen who were not in a position to pay the expenses of private nursing might be aided in obtaining treatment. It was agreed to appoint a committee, and Sir W. Lethbridge, Surgeon-Major General Sir William Hooper, Mr. Wolfenden, and Mr. Praed were elected.

In the course of a lecture at the Institute of Hygiene, on the 27th inst., Dr. N. Meachen laid great emphasis upon the universal necessity for regular and systematic exercise. The lecturer, we believe, intended his views mainly, if not entirely, for those who have passed the age of infancy. But in America there appears to be a school which advocates that babies from a few weeks old and onwards should be studiously drilled in gymnastic performances. An enthusiastic advocate of this treatment states that a healthy baby "will naturally take to exercise of a physical-culture character and enjoy it hugely."

